# Effectiveness of Live-Attenuated Genotype III Japanese Encephalitis Viral Vaccine against Circulating Genotype I Viruses in Swine

**DOI:** 10.3390/v14010114

**Published:** 2022-01-09

**Authors:** Yi-Chin Fan, Yi-Ying Chen, Jo-Mei Chen, Chienjin Huang, Mei Huang, Shyan-Song Chiou

**Affiliations:** 1 Graduate Institute of Microbiology and Public Health, National Chung Hsing University, Taichung 402, Taiwan; yichinfan@ntu.edu.tw (Y.-C.F.); irisgf1986@gmail.com (Y.-Y.C.); chenjm0317@gmail.com (J.-M.C.); cjhuang@dragon.nchu.edu.tw (C.H.); 2 Institute of Epidemiology and Preventive Medicine, College of Public Health, National Taiwan University, Taipei 10617, Taiwan; 3 Division of Infectious Disease, Chang Bing Show Chwan Memorial Hospital, Changhua 505, Taiwan

**Keywords:** Japanese encephalitis virus, genotype, live-attenuated vaccine, vaccine effectiveness, swine

## Abstract

Expansion of genotype I (GI) Japanese encephalitis viruses (JEV) has resulted in the replacement of the dominant genotype III (GIII) viruses, raising serious public health concerns for using GIII virus-derived vaccines to effectively control JEV epidemics. Therefore, this study used swine as the model to estimate the effectiveness of GIII live-attenuated vaccine against GI virus infection by comparing the incidence of stillbirth/abortion in gilts from vaccinated and non-vaccinated pig farms during the GI-circulation period. In total, 389 and 213 litters of gilts were recorded from four vaccinated and two non-vaccinated pig farms, respectively. All viruses detected in the aborted fetuses and mosquitoes belonged to the GI genotype during the study period. We thus estimated that the vaccine effectiveness of GIII live-attenuated vaccine against GI viruses in naive gilts based on the overall incidence of stillbirth/abortion and incidence of JEV-confirmed stillbirth/abortion was 65.5% (50.8–75.7%) and 74.7% (34.5–90.2%), respectively. In contrast to previous estimates, the GIII live-attenuated vaccine had an efficacy of 95.6% (68.3–99.4%) to prevent the incidence of stillbirth/abortion during the GIII-circulating period. These results indicate that the vaccine effectiveness of GIII live-attenuated JEV vaccine to prevent stillbirth/abortion caused by GI viruses is lower than that against GIII viruses.

## 1. Introduction

Japanese encephalitis (JE), caused by Japanese encephalitis virus (JEV) infection, is an important zoonotic viral disease in East/Southeast/South Asia [1,2]. JEV circulates among amplifying hosts, including swine, birds, and Culex mosquitoes, mainly *Culex tritaeniorhynchus* [1]. Humans and other affected domestic animals (such as goats and horses) are dead-end hosts in the JEV transmission cycle because of the low level and short duration of viremia [3]. Vaccination is the most effective prevention strategy for JE, and only licensed GIII-based vaccines are available for use in humans and swine [4,5]. As Taiwan is in a JEV endemic region, residential children are routinely vaccinated with four doses of inactivated JEV vaccine or two doses of 17D-JEV chimeric vaccine, whereas swine gilts receive two doses of live-attenuated vaccine [6,7].

JEV is a member of the flavivirus family, and contains a single-stranded, positive-sense RNA genome encoding three structural proteins, capsid (C), membrane (M), and envelope (E). The multifunctional E protein is the primary target for eliciting protective immunity [8]. Based on JEV E protein sequence analysis, viruses can be classified into five genotypes (I, II, III, IV, and V) that originate from the Indonesia/Malaysia region and expand and circulate in East/Southeast/South Asian countries. Before the 1990s, GIII was the most widely distributed and frequently isolated in JE epidemic regions [9]. Therefore, all licensed JEV vaccines, inactivated or live-attenuated, and for humans or swine, are GIII-derived [4], including the live-attenuated SA14-14-2, inactivated Nakayama, and yellow fever 17D-SA14-14-2 live-chimeric vaccine for humans, as well as the live-attenuated ML-17 or AT222 for swine. However, the expansion of GI viruses, including Ia and Ib, has been observed since the 1990s and these have gradually replaced GIII viruses as the dominant circulating viruses in JE epidemic regions [10]. The mechanism of this JEV genotype replacement, associated with the enhanced infectivity of GI viruses in amplifying hosts (swine and avian), is caused by a combination of amino acid substitutions in the NS2B/NS3 or NS5 proteins [11,12].

The E protein has approximately 12% nucleotide and 3% amino acid sequence differences between GI and GIII JEVs [6]. Thus, it is important to understand the heterologous protection of GIII-derived JE vaccines against GI viruses. Currently, many studies have used in vitro seroprotection studies to estimate the effectiveness of GIII-based vaccines. GIII-inactivated JEV vaccines administered to travelers were found to elicit seroprotective levels of neutralizing antibodies (≥1:10 dilution) against heterologous GI viruses when the serum was collected 4–8 weeks after vaccination; however, compared to the GIII vaccine strain, the neutralizing titers against GI viruses were 6- to 10-fold lower [13]. Among the vaccinated residents in JEV-endemic regions, the serum collected at various times after vaccination showed an 8-fold higher neutralizing antibody titer against the GIII vaccine strain than that against GI viruses, and the seroprotective period estimated for GI viruses was much shorter than that for GIII JEV [14]. Similarly, serum collected from sows at 4 weeks after primary immunization showed a 32-fold higher neutralizing antibody titer against the GIII vaccine strain than against the GI viruses [6].

Although several reports have indicated high homologous effectiveness [7,15,16], the heterologous effectiveness of GIII-derived JE vaccines against GI viruses has not been determined to date. JEV infection is the major cause of abortion/stillbirth in pregnant sow [1,17,18]. Therefore, in the current study, we assessed the heterologous effectiveness of GIII-derived, live-attenuated JE vaccines against GI viruses using a pig model by analyzing the incidence of stillbirth/abortion among vaccinated and unvaccinated gilts. This field study provided the first evidence of effectiveness/efficacy of GIII vaccine against GI JEV in swine.

## 2. Materials and Methods

### 2.1. Study Design

**Figure 1:**
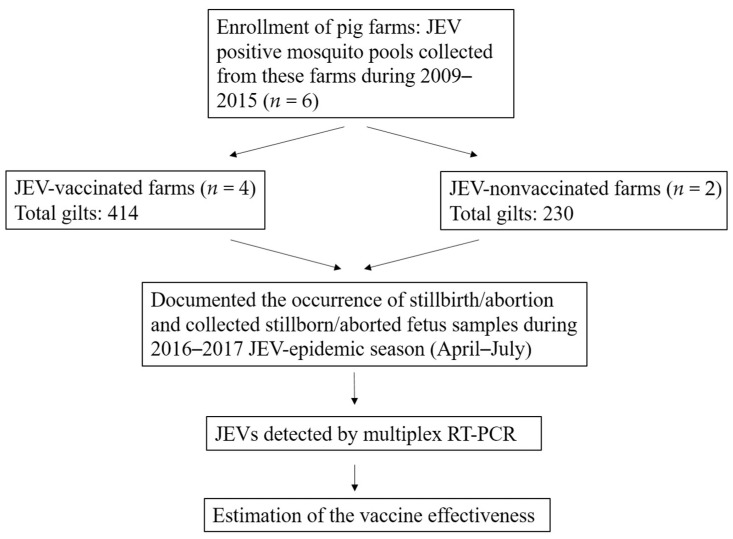
Flow chart of the vaccine effectiveness study.

Since the replacement of the JEV GIII with GI in Taiwan in 2008–2009, virological surveillance has been persistently conducted in pig farms [6,19]. In this study, JEV-circulating pig farms, at which JEV has been detected in the mosquitoes collected from these farms during 2009–2015, were enrolled in the study (Figure 1). To prevent stillbirth/abortion, the gilts (virgin and chosen to conceive female pig) received two doses of the GIII live-attenuated JEV AT222 strain vaccine in Taiwan. There was no intervention in the decision of farm owners on whether to vaccinate against JEV in this study. Four of the enrolled farms implemented vaccination, and two farms were non-vaccination.

Studies conducted since 1976, The JEV-specific neutralizing antibodies was higher than 50% annually in non-vaccinated pigs’ serum specimens collected from slaughterhouses and pig farms. Thus, JEV is actively transmitted in pig farms in Taiwan, and more than 50% of pigs are infected annually [6,7]. To prevent the confounding effect on vaccine effectiveness estimation owing to previous JEV infection, the pregnancy status of the first litter of the gilts was recorded. During the 2016–2017 JEV-epidemic season, from April to July in Taiwan, the occurrence of stillbirth/abortion was recorded by active surveillance through telephone inquiry every morning, and two stillborn/aborted fetuses were collected from each litter. This field study protocol was approved by the Institutional Animal Care and Use Committee of National Chung Hsing University (Protocol No: 108–117).

### 2.2. Sample Collection

The stillborn/aborted fetuses were placed in an ice box and shipped to the laboratory every morning. Brain tissue (5–10 g section) was aseptically collected from dissected fetuses, placed in collection tubes, and stored at −80 °C. Blood samples from the sows (non-virgin female pigs) and gilts were collected before and after immunization, serum specimens were obtained by centrifugation, stored at −80 °C for JEV-specific neutralizing antibody assay which performed in the same year of collection.

### 2.3. Neutralizing Antibody

A focus-reduction microneutralization titer (FRμNT) assay was used to measure the neutralizing antibodies in serum specimens. First, Vero cells were seeded in 96-well plates and incubated for 20 h in a 37 °C incubator with 5% CO_2_. Inactivated serum specimens were serially diluted and incubated with 100 focus forming units (ffu) of JEV at 37 °C for 1 h. The monolayer of Vero cells in the 96-well plate was infected with the serum-virus mixture at 37 °C for 1 h. The infected cells were overlaid with 1% methyl cellulose and 2% FBS in DMEM and incubated in a 37 °C incubator with 5% CO_2_ for 30 h. The infected cells were then washed with PBS to remove 1% methyl cellulose, fixed with 75% acetone in PBS at room temperature for 20 min, and dried under a hood. The fixed cells were incubated with mouse anti-JEV polyclonal antibody at 37 °C for 40 min, and JEV-reactive mouse antibodies were detected by incubating with peroxidase-conjugated goat anti-mouse IgG (H + L) antibodies (Jackson ImmunoResearch, West Grove, PA, USA) at 37 °C for another 40 min. The virus-infected foci were counted after staining with the Vector-VIP peroxidase substrate kit SK-4600 (Vector Laboratories, Burlingame, CA, USA). The cut-off value of FRμNT_50_ was at the 1:10 serum dilution, and the foci count reduced by at least 50% of that in the virus-only control well.

### 2.4. Multiplex RT-PCR

Total viral RNA was extracted from the homogenate mosquito and brain tissue of a swine fetus sample using a viral RNA extraction kit (Viogene Biotek Corp., Taipei, Taiwan). cDNA was synthesized using Superscript^®^III reverse transcriptase (Invitrogen, Houston, TX, USA) with random primers at 50 °C for 1 h, and the products were stored at −20 °C for later use.

Multiplex RT-PCR was performed in a 25 µL mixture containing 12.5 µL GoTaq^®^ Master Mix (Promega, Madison, WI, USA), 10 µM of JEV universal and GI- and GIII-specific primers, and 4 µL cDNA; the mixture was brought up to 25 µL by with ddH_2_O. The amplification program comprised one cycle at 94 °C for 5 min, followed by 30 cycles at 94 °C for 30 s, 50 °C for 1 min, and 72 °C for 1 min, followed by termination with a final extension step of 7 min at 72 °C. The PCR products were separated by electrophoresis on 2% agarose gels and were analyzed after staining with HealthView Nucleic Acid Stain (Genomics, Taipei, Taiwan).

### 2.5. Phylogenetic Analysis

Nucleotide sequences were assembled using SeqMan II software (version 5.03; DNASTAR; https://www.dnastar.com/; accessed on 25 August 2021), and were aligned using the Clustal W multiple alignment tool in the BioEdit Sequence Alignment Editor (version 7.0.9.0; https://bioedit.software.informer.com/7.0/; accessed on 9 March 2017). The phylogenetic tree was inferred based on the 1500-nucleotide sequence of the full-length JEV envelope gene. A phylogenetic tree was constructed using the maximum likelihood method with 1000 bootstrap replicates in MEGA X software, version 10.2.6 (https://www.megasoftware.net/; accessed on 9 November 2021).

### 2.6. Statistics

Two incidences were calculated. The incidence rate of stillbirth/abortion was calculated using the recorded data per 100 litters at risk, and the incidence rate of JEV-related stillbirth/abortion was calculated using the positivity rate of multiplex RT-PCR per 100 aborted fetuses at risk. Vaccine effectiveness (VE) was estimated as one minus the relative risk (RR = incidence of vaccinated group divided by incidence of unvaccinated group) between the two groups of interest (VE = 1 − RR). Statistical significance was set at *p* < 0.05. All statistical analyses were performed using SAS 9.2 statistical software package (SAS Institute Inc., Cary, NC, USA).

## 3. Results

### 3.1. Characteristics of Enrolled Pig Farms

Pig farms used for active JEV surveillance between 2009–2015 were enrolled in the study [6,19,20]. In 2015, a total of six pig farms were eligible based on four criteria: status of JEV circulation, farming type (farrow-to-finish or matching breed), status of JEV vaccination, and owner’s agreement (Figure 1 and Table 1). These farms were in central and southern Taiwan; four farms belonged to the farrow-to-finish type with 36–56 gilts, and two farms belonged to matching breed type with more than 150 gilts. The gilts (virgin and reproductive active female pigs) from four farms (A–D) received two doses of primary vaccination with GIII live-attenuated JEV AT222 strain vaccines through subcutaneous or intramuscular routes from January to March, before the JEV epidemic season (April to July).

In 2016, serum samples from 10 sows (nonvirgin and reproductive female pigs) and gilts were collected from each farm, and neutralizing antibodies determined against GI and GIII JEVs (Table 2). The positivity of neutralizing antibodies against GI and GIII among the sows of each farm was higher than 80% owing to vaccination or natural infection. Among gilts, only three samples showed low antibody titers against GIII viruses (data not shown) before vaccination, but all samples were positive after vaccination; no sample was positive against GI virus before vaccination and only 30–40% were antibody positive against GI virus after vaccination. Based on these results, the gilts were selected as a model to estimate vaccine effectiveness to prevent confusion regarding antibody titers from natural JEV infection and vaccination.

### 3.2. Occurrence of Stillbirth/Abortion

During the 2016–2017 JEV-transmission season, stillbirth/abortion in the six enrolled pig farms was confirmed and recorded every morning through an active telephone conference call. Two stillborn/aborted fetuses from each litter were collected and dissected, and the brain tissue of each aborted fetus was collected (Table 3). In total, 41 of 389 (10.54%) and 65 of 213 (30.52%) litters were stillborn/aborted among four vaccinated and two non-vaccinated pig farms, respectively (*p* < 0.05).

**Figure 2:**
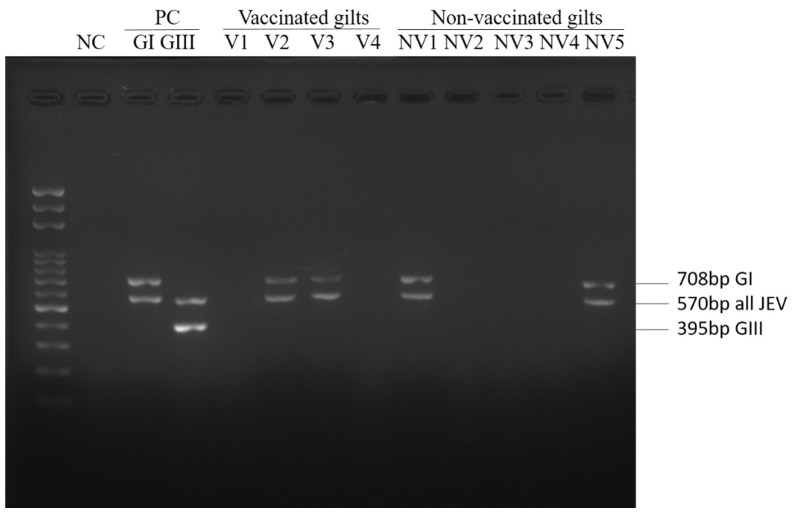
Multiplex RT-PCR results of brain specimen of stillbirth/aborted fetuses. The multiplex RT-PCR comprises three specific primers sets, the JEV-specific, GI-specific, and GIII-specific primer sets yielding 570 bp, 708 bp, and 395 bp DNA products, respectively [21].

The collected brain tissue of aborted fetuses was subjected to JEV genotyping using a multiplex RT-PCR assay [21] to determine the infected genotype causing stillbirth/abortion (Figure 2). All JEV-positive tested specimens showed the 708 and 570 base-pair DNA products and were classified as having GI infection. Eight JEV-positive specimens belonging to six litters were collected from three JEV-vaccinated farms (except Farm A), and 19 positive specimens belonging to 13 litters were collected from two JEV non-vaccinated farms (Table 3 and Table S1). Thus, six of 389 (1.54%) and 13 of 213 (6.10%) stillborn/aborted litters were JEV-confirmed among the JEV-vaccinated and non-vaccinated pig farms, respectively (*p* < 0.05).

**Figure 3:**
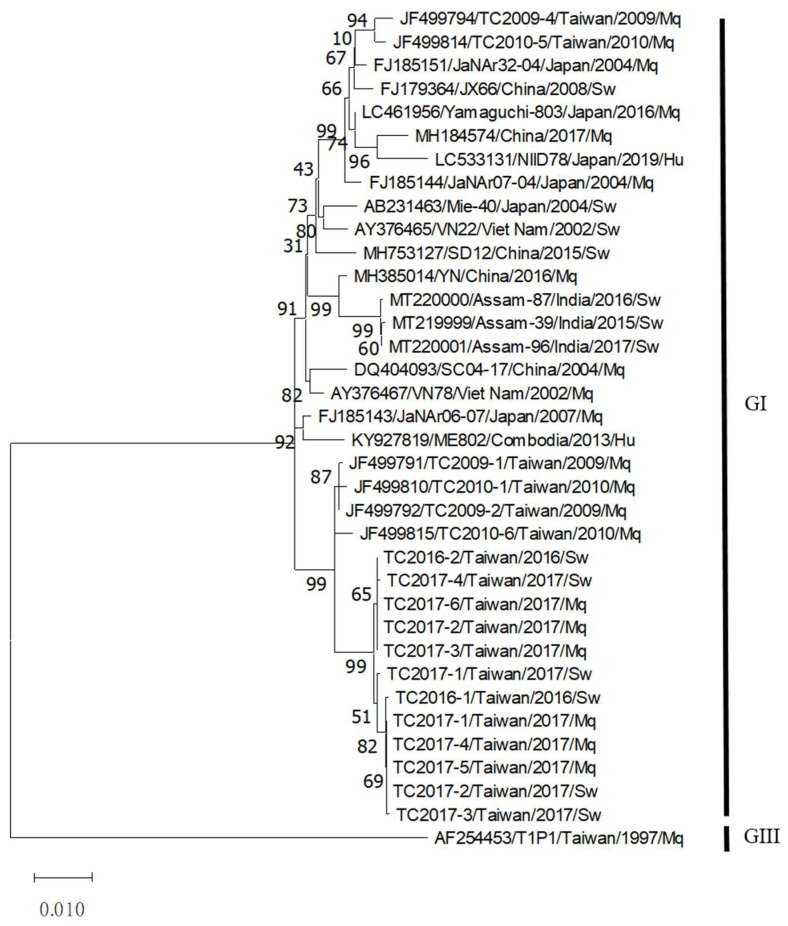
Phylogenetic analysis of JEV detected in this study. Using the GIII JEV T1P1 strain as the root, a phylogenetic tree was constructed based on the full-length E protein sequence using the maximum likelihood method with 1000 bootstrap replicates.

During the study period, mosquitoes were also collected from the study farms and subjected to JEV genotyping using a multiplex RT-PCR assay. The full-length E sequences of JEV-positive mosquitoes and aborted fetuses were then obtained and subjected to phylogenetic analysis (Figure 3 and Table S2). The sequences of all JEV-positive samples collected from the mosquito and aborted fetus specimens belonged to the same cluster of GI virus sequences during the study period.

### 3.3. Estimates of Vaccine Effectiveness

The effectiveness of the GIII vaccine against stillbirth/abortion caused by G1 infection was estimated in vaccinated and non-vaccinated gilts. During the GIII circulating period, the relative risk (vaccination vs. non-vaccination) was 0.044 [(1/74)/(21/68)] and vaccine efficacy was 95.6% (68.3–99.4) [15]; in contrast, during the GI circulation period,, the relative risk was 0.345 [(41/389)/(65/213)] and vaccine effectiveness was 65.5% (50.8–75.7) (Table 4). However, the stillbirth/abortion of gilts was not solely caused by JEV infection. Thus, based on the incidence of JEV-confirmed stillbirth/abortion to evaluate the effectiveness of the live-attenuated GIII JEV vaccine, the relative risk was 0.253 [(6/389)/(13/213)] and vaccine effectiveness was 74.7% (34.5–90.2) during the GI circulation period, as determined in this study; however, vaccine effectiveness data using JEV-confirmed stillbirth/abortion during the GIII circulation period were unavailable.

## 4. Discussion

JEV outbreaks caused by circulating viruses have shifted from GIII and have been dominated by GI viruses in the past decades because of the replication advantage of GI viruses in the amplifying swine and avian hosts [11,12]. The consequences of JEV genotype replacement on the ecology, pathogenicity, and vaccination have been reported. Both genotypes are frequently isolated from Culex mosquitoes [19,22] and from swine [23,24]. The similar infectivity and transmission ability of GI and GIII JEVs in Culex mosquitoes [25], and the similar incidence of infection in humans [26] indicate that the ecology of JEV remains unchanged after genotype replacement. Both genotypes have a similar 50% lethal dose of infection in a mouse model [27] and the symptomatic/asymptomatic ratio in humans [26] suggests that both genotypes are equally pathogenic. Several studies have investigated the effect of JEV genotype replacement on the effect of GIII vaccination using mouse and swine models and serum specimens from GIII-vaccinated human individuals, but the results were inconsistent [6,13,14,28,29,30,31,32]. However, the titers of neutralizing antibodies elicited by GIII vaccines were consistently lower against GI viruses than those against GIII wild-type viruses and GIII-vaccine strains [6,13,14].

The GIII vaccine-immunization conferred partial protection against GI virus challenge in mice, but provided complete protection against GIII viruses [28]. For many years, the consequences of JEV genotype replacement on vaccination in target hosts such as humans, and swine, were based on the measurement of the cross-neutralizing activity of antibodies elicited by vaccination, but not on protection. Limited data are available to estimate the cross-genotype effectiveness of JEV vaccines because of the low incidence of confirmed cases among the human population, confounded by the protection elicited via natural infection, uncertain circulating genotype in the defined period, and others. We took advantage of the high annual JEV infection rate (more than 50%) in the swine population during the outbreak season in Taiwan [6] and found that JEV infection is the major cause of stillbirth/abortion in non-immune gilts. We estimated vaccine effectiveness using stillbirth/abortion in gilts (not sows that were vaccinated and exposed to natural virus infection during the past outbreak) as an outcome measure to avoid the confounding effect of previous infection. We also analyzed the JEV genotype in aborted fetal brain tissues to determine the genotype and transmission activity during the study period [19,22].

In general, the reproductively active sow produces litters twice a year, and the rate of stillbirth/abortion in pregnant sows caused by porcine reproductive and respiratory syndrome virus, pseudorabies virus, JEV, and porcine circoviruses is approximately 23.6% in Taiwan (Official report of the Council of Agriculture, Taiwan). Porcine reproductive and respiratory syndrome virus, pseudorabies virus, and porcine circoviruses are included in mass immunization programs in Taiwan, thus biased effect of these pathogens on JE vaccine effectiveness estimation could be minimized. To reduce the confounding effect in sows naturally exposed to JEV infection, only the first litter of gilts was recorded and used in this study (Table 3). The stillbirth/abortion rate in JEV-vaccinated pig farms was 10.54% (41/389) and of these, 14.63% (6/41) were attributed to JEV infection; among non-vaccinated pig farms, 30.52% (65/213) and 20% (13/65) of these were attributed to JEV infection.

Serological evidence revealed that the homologous neutralizing activity of GIII vaccines was significantly higher than that of heterologous viruses, especially against GI viruses in swine models. In serum collected from pigs at 4 weeks after receiving two doses of live-attenuated GIII JEV vaccine, the neutralizing antibody titer was 32-fold higher against the GIII vaccine strain than that against GI viruses [6]. Similarly, the positivity rate of neutralizing antibodies in vaccinated gilts was lower against GI viruses than that against GIII viruses (Table 2). Consistent with heterologous cross-neutralizing activity, the effectiveness of GIII live-attenuated JEV vaccine in gilts against GIII viruses (95.6%) was higher than that against GI viruses (65.5% or 74.7%), using either the incidence of stillbirth/abortion or JEV-confirmed stillbirth/abortion in this study (Table 4).

Among vaccinated children, the vaccine effectiveness of the inactivated GIII JEV vaccine was reported to range from 86.56–96.98% in Taiwan [7,16]. The presumptive protective threshold of antibodies elicited by the inactivated GIII JEV vaccine against the GI viruses among children was 1:80 (titer against Nakayama vaccine strain) [14]; and the presumptive protective threshold of antibodies elicited by the live-attenuated GIII JEV vaccine among swine was 1:320 (against AT222 vaccine strain) [6]. Thus, we could expect that the protective effectiveness of GIII inactivated JEV vaccine against GI viruses in vaccinated children might be higher than 65.5% or 74.7% as revealed in swine in this study, but lower than 86.56–96.98%, as estimated from previous studies in vaccinated children against GIII viruses in Taiwan [7,16].

We recognize that there are potential limitations to this study including the different JEV activity between pig farms, herd immunity derived from vaccinated and/or naturally infected sows blocking the JEV transmission in farms, and the noncurrent comparison of vaccine effectiveness against GI and GIII viruses. The enrolled farms were selected based on the presence of active JEV circulation in previous studies; JEV circulation was also detected among the participating farms, except for Farm A, during the study period. JEV immune sows constituted a small fraction (less than 10%) of the total pigs; thus, herd immunity should not have a major effect of interrupting JEV transmission in the study farms. Currently, GI and GIII viruses are co-circulating in China and India [33,34,35,36]. It might thus be impractical to concurrently estimate vaccine efficacies against GI and GIII viruses in these regions owing to the dynamic and mutual interference of genotype transmission.

GI JEV has rapidly replaced the GIII viruses and has become the dominant genotype in most endemic Asian countries since the 1990s [37]. JEV vaccination programs are the most effective control measures to reduce the impact of epidemics or endemic viruses in affected countries [5]. All currently available JEV vaccines are derived from GIII viruses [38,39]. Our current results using gilts vaccinated with live-attenuated GIII vaccine as the model showed that the effectiveness of the GIII vaccine against circulating GI viruses was lower compared to that reported in previous studies on GIII viruses. Further efforts are thus needed to improve the immunogenicity of current vaccines or to develop new GI-specific vaccines to prevent stillbirth/abortion in pigs according to a cost/benefit analysis.

## Figures and Tables

**Table 1:** Characteristics of pig farms enrolled in this study.

Characteristic	Pig Farms					
	A	B	C	D	E	F
JEV circulating *	Yes	Yes	Yes	Yes	Yes	Yes
Location of Taiwan	Central	Central	Southern	Southern	Central	Southern
No. of gilts **	36	50	56	272	54	176
Type of farm	farrow-to-finish	farrow-to-finish	farrow-to-finish	matching breed	farrow-to-finish	matching breed
JEV Vaccination	Yes	Yes	Yes	Yes	No	No
Type of Vaccine	Attenuated	Attenuated	Attenuated	Attenuated		
Time of vaccination	Feb-Mar	Jan-Feb	Feb-Mar	Feb-Mar		
Route of vaccination	subcutaneous	intramuscular	subcutaneous	intramuscular		
Dosage	2	2	2	2		

* [6,19,20]. ** Two years (2016 and 2017).

**Table 2:** Neutralizing antibody against GI and GIII JEVs in serum samples collected from sows and gilts in 2016.

Positivity of Neutralizing Antibody (%)					
Against GIII JEV			Against GI JEV		
Sow	Gilt (Before Vaccination)	Gilt (After Vaccination)	Sow	Gilt (Before Vaccination)	Gilt (After Vaccination)
80	10	100	80	0	40
100	0	100	90	0	40
80	0	100	80	0	30
90	10	100	90	0	40
90	0	0	90	0	0
80	10	0	80	0	0

**Table 3:** JEV detection among stillborn/aborted fetus samples collected from GIII live-attenuated JEV-vaccinated and non-vaccinated gilts during the 2016–2017 JEV-epidemic season.

JEV Vaccination	Vaccination	Non-Vaccination
Farms	4	2
Gilts	414	230
Litter	389	213
Stillbirth-abortion	41	65
Samples of Stillbirth/abortion	82	130
Positive of JEV multiplex RT-PCR		
GI	8(6) *	19(13) **
GIII	0	0

* Eight JEV-positive samples belonged to six litters. ** 19 JEV-positive samples belonged to 13 litters.

**Table 4:** Estimation of the effectiveness of GIII live-attenuated JEV vaccine against GI and GIII viruses in gilts.

Study Period	Circulating JEV Genotype	Vaccination Status of Gilt	Incidence (Abortion * or JEV(+) **)	Relative Ratio	Vaccine Effectiveness (%)	Reference
2016–2017	GI	Yes	41/389 *	0.345 (0.243–0.492)	65.5 (50.8–75.7)	This study
		No	65/213 *			
		Yes	6/389 **	0.253 (0.098–0.655)	74.7 (34.5–90.2)	This study
		No	13/213 **			
1969–1970	GIII	Yes	1/74 *	0.044 (0.006–0.316)	95.6 (68.3–99.4)	[15]

* Incidence of stillbirth/abortion. ** Incidence of JEV-confirmed stillbirth/abortion.

## Data Availability

All data generated and analyzed during this study are included in this article and its supplementary information files.

## Supplementary Materials

The following are available online at https://www.mdpi.com/article/10.3390/v14010114/s1, Table S1: list of JEV-positive aborted fetus, Table S2: list of the full-length E sequences of JEV-positive mosquitoes and aborted fetuses obtained in this study.
