# Supplementary material for: Effectiveness of Live-Attenuated Genotype III Japanese Encephalitis Viral Vaccine against Circulating Genotype I Viruses in Swine
Source: Viruses. 2022 Jan 9;14(1):114. doi: 10.3390/v14010114 (PMC8778556; doi:10.3390/v14010114)
Supplement: Supplementary file 1 [file viruses-14-00114-s001.zip › viruses-1521125-supplementary.pdf]

Supplementary Materials

Table S1. List of JEV-positive aborted fetus

Farm	JEV vaccination	Date of abortion	JEV genotype	Remark
B	Yes	2017.04.27	I	
C	Yes	2017.05.23	I	
D	Yes	2016.04.02	I	
D	Yes	2017.05.14	I	Same litter
D	Yes	2017.05.14	I	
D	Yes	2017.05.30	I	
D	Yes	2017.06.06	I	Same litter
D	Yes	2017.06.06	I	
E	No	2016.04.11	I	
E	No	2017.04.11	I	Same litter
E	No	2017.04.11	I	
E	No	2017.04.30	I	
E	No	2017.05.01	I	Same litter
E	No	2017.05.01	I	
E	No	2017.05.04	I	
F	No	2016.05.17	I	Same litter
F	No	2016.05.17	I	
F	No	2016.05.18	I	
F	No	2017.04.26	I	
F	No	2017.05.09	I	Same litter
F	No	2017.05.09	I	
F	No	2017.05.09	I	Same litter
F	No	2017.05.09	I	
F	No	2017.05.10	I	Same litter
F	No	2017.05.10	I	
F	No	2017.05.10	I	
F	No	2017.05.12	I	

Table S2. List of the full-length E sequences of JEV-positive mosquitoes and aborted fetuses obtained in this study.

Name	Farm	Time	Host	Genotype	Accession no.
TC2016-1/Taiwan/2016/Sw	D	2016.04.02	Swine	I	MZ733971
TC2016-2/Taiwan/2016/Sw	F	2016.05.17	Swine	I	MZ733972
TC2017-1/Taiwan/2017/Sw	D	2017.06.06	Swine	I	MZ733961
TC2017-2/Taiwan/2017/Sw	E	2017.05.01	Swine	I	MZ733962
TC2017-3/Taiwan/2017/Sw	F	2017.05.09	Swine	I	MZ733963
TC2017-4/Taiwan/2017/Sw	F	2017.05.12	Swine	I	MZ733964
TC2017-1/Taiwan/2017/Mq	A	2017.05.05	*Cx. tritaeniorhynchus*	I	MZ733965
TC2017-2/Taiwan/2017/Mq	C	2017.05.05	*Cx. tritaeniorhynchus*	I	MZ733966
TC2017-3/Taiwan/2017/Mq	D	2017.05.29	*Cx. tritaeniorhynchus*	I	MZ733967
TC2017-4/Taiwan/2017/Mq	E	2017.05.06	*Cx. tritaeniorhynchus*	I	MZ733968
TC2017-5/Taiwan/2017/Mq	E	2017.05.06	*Cx. tritaeniorhynchus*	I	MZ733969
TC2017-6/Taiwan/2017/Mq	F	2017.05.10	*Cx. tritaeniorhynchus*	I	MZ733970
